# Multiplexed immunohistochemical evaluation of small bowel inflammatory and epithelial parameters in environmental enteric dysfunction

**DOI:** 10.1016/j.ajcnut.2024.02.033

**Published:** 2024-09-17

**Authors:** Kelley VanBuskirk, Monica Mweetwa, Tad Kolterman, Shyam Raghavan, Tahmeed Ahmed, S Asad Ali, SM Khodeza Nahar Begum, Ellen Besa, Donna M Denno, Zehra Jamil, Paul Kelly, Mustafa Mahfuz, Sean R Moore, Samer Mouksassi, William A Petri, Phillip I Tarr, Peter B Sullivan, Christopher A Moskaluk, EEDBI Consortium, Kumail Ahmed, Sheraz Ahmed, Md Ashraful Alam, Subhasish Das, Lee A Denson, Shah Mohammad Fahim, Md Amran Gazi, Yael Haberman, Md Mehedi Hasan, Md Shabab Hossain, Aneeta Hotwani, Junaid Iqbal, Najeeha Talat Iqbal, Sadaf Jakhro, Furqan Kabir, Ta-Chiang Liu, Barbara J Mann, Chelsea Marie, Ramendra Nath Mazumder, Victor Mudenda, Chola Mulenga, Abdul Khalique Qureshi, Masudur Rahman, Najeeb Rahman, Kamran Sadiq, Guillermo J Tearney, Fayaz Umrani, Omer H Yilmaz

**Affiliations:** 15 Department of Paediatrics and Child Health, Aga Khan University, Karachi, Pakistan; 16 Nutrition Research Division, International Centre for Diarrhoeal Disease Research, Bangladesh, Dhaka, Bangladesh; 17 Division of Pediatric Gastroenterology, Hepatology, and Nutrition, Cincinnati Children's Hospital, Medical Center, Cincinnati, OH, USA; 18 Department of Pathology and Immunology, Washington University School of Medicine, St. Louis, MO, USA; 19 Department of Medicine, University of Virginia School of Medicine, Charlottesville, VA, USA; 20 Tropical Gastroenterology & Nutrition group, University of Zambia School of Medicine, Lusaka, Zambia; 21 Department of Gastroenterology, Sheikh Russel National Gastroliver Institute and Hospital, Dhaka, Bangladesh; 22 Department of Pathology, Harvard Medical School, Boston, MA, USA; 23 Department of Pathology, Massachusetts General Hospital, Boston, MA, USA; 1 Department of Global Health, University of Washington School of Public Health, Seattle, WA, United States; 2 Tropical Gastroenterology and Nutrition Group, University of Zambia School of Medicine, Lusaka, Zambia; 3 Department of Pathology, University of Virginia School of Medicine, Charlottesville, VA, United States; 4 Nutrition Research Division, International Centre for Diarrhoeal Disease Research, Bangladesh, Dhaka, Bangladesh; 5 Department of Paediatrics and Child Health, Aga Khan University, Karachi, Pakistan; 6 Department of Pathology, Bangladesh Specialized Hospital, Dhaka, Bangladesh; 7 Department of Pediatrics, University of Washington School of Medicine, Seattle, WA, United States; 8 Department of Biological and Biomedical Sciences, Aga Khan University, Karachi, Pakistan; 9 Blizard Institute, Barts & the London School of Medicine, Queen Mary University of London, London, United Kingdom; 10 Department of Pediatrics, University of Virginia School of Medicine, Charlottesville, VA, United States; 11 Certara, Princeton, NJ, United States; 12 Department of Medicine, University of Virginia School of Medicine, Charlottesville, VA, United States; 13 Department of Pediatrics, Washington University School of Medicine, St. Louis, MO, United States; 14 Department of Paediatrics, Children’s Hospital, University of Oxford, Oxford, United Kingdom

**Keywords:** environmental enteric dysfunction, enteropathy, immunohistochemistry, gene expression, histopathology, histology, cellular differentiation

## Abstract

**Background:**

Environmental enteric dysfunction (EED) is characterized by reduced absorptive capacity and barrier function of the small intestine, leading to poor ponderal and linear childhood growth.

**Objectives:**

To further define gene expression patterns that are associated with EED to uncover new pathophysiology of this disorder.

**Methods:**

Duodenal biopsies from cohorts of children with EED from Bangladesh, Pakistan and Zambia were analyzed by immunohistochemistry (IHC) to interrogate gene products that distinguished differentiation and various biochemical pathways in immune and epithelial cells, some identified by prior bulk RNA sequence analyses. Immunohistochemical staining was digitally quantified from scanned images and compared to cohorts of North American children with celiac disease (gluten-sensitive enteropathy) or with no known enteric disease and no pathologic abnormality (NPA) detected in their clinical biopsies.

**Results:**

After multivariable statistical analysis, we identified statistically significant (*P* < 0.05, 2-tailed t-test) elevated signals representing cluster of differentiation 45 (80%; 95% confidence interval [CI]: 24%, 127%), lipocalin 2 (659%; 95% CI: 198%, 1838%), and regenerating family 1 beta (221%; 95% CI: 47%, 600%) and lower signals corresponding to granzyme B (−74%; 95% CI: −82%, −62%), and sucrase isomaltase (−58%; 95% CI: −75%, −29%) in EED biopsies compared with NPA biopsies. Computerized algorithms also detected statistically significant elevation in intraepithelial lymphocytes (49%; 95% CI: 9%, 105%) and proliferation of leukocytes (267%; 95% CI: 92%, 601%) in EED biopsies compared with NPA biopsies.

**Conclusions:**

Our results support a model of chronic epithelial stress that decreases epithelial differentiation and absorptive function. The close association of several IHC parameters with manual histologic scoring suggests that automated digital quantification of IHC panels complements traditional histomorphologic assessment in EED.

## Introduction

Environmental enteric dysfunction (EED) is a highly prevalent condition in low- and middle-income countries (LMICs) that is characterized by reduced absorptive capacity and barrier function of the small intestine, leading to poor ponderal and linear childhood growth [1]. Malabsorptive syndromes have been described in local and expatriate residents of “tropical” areas, with intestinal inflammation and altered mucosal architecture. This may be symptomatic, such as tropical sprue [2] or asymptomatic, such as tropical enteropathy [3] or environmental enteropathy [4,5]. The term EED refers to the asymptomatic intestinal dysfunction associated with exposure to environments with poor sanitation and hygiene rather than tropical geography per se [6,7] but encompasses the previously mentioned syndromes. Histologic changes of small intestinal mucosa in these disorders include blunting or complete atrophy of villus architecture, chronic inflammation in the lamina propria, and elevated numbers of intraepithelial lymphocytes (IELs) [[8], [9], [10], [11], [12]]. In addition to histologic changes, altered gut function in EED includes malabsorption [13,14], decreased barrier function [15,16], and diminished immune response to oral vaccination [17,18].

Recent studies have begun to characterize the molecular changes in the gut mucosa of individuals with EED, including RNA transcriptomics [[19], [20], [21]]. Such studies have identified major differences in gene expression and potential pathways that are dysregulated in EED. However, because these studies were performed on whole tissue biopsies, the exact tissue constituents in which these gene expression alterations lie are for the most part still unknown. The technique of immunohistochemistry (IHC), which employs antibody binding to antigens in specific gene products, is commonly used to identify the location of these gene products in specific cellular constituents of normal and diseased tissue. To further define the pathophysiology of EED, we now report the findings from several panels of immunohistochemical stains in a large cohort of endoscopic biopsies of children with suboptimal growth in 3 LMICs (Bangladesh, Pakistan, and Zambia).

## Methods

### Participants and tissue samples

The participant cohorts’ enrollment criteria, study procedures, consenting procedures, and study time frame are shown in Supplementary Table 1 and are described in a companion paper [22]. Informed consent from all participants and/or their guardians was obtained, and research protocols at all participating institutions were evaluated and approved by ethics boards/institutional review committees. Duodenal biopsies obtained from participants in Bangladesh, Pakistan, and Zambia were from low-income, socially disadvantaged communities and fulfilled criteria for suboptimal growth despite nutritional supplementation. Each of the 3 centers used community-based enrollment to identify children meeting their anthropometry-based criteria to receive nutritional intervention. Bangladeshi children were enrolled based on length-for-age Z score (LAZ), falling into 2 groups: stunted (LAZ <−2) and “at risk for stunting” (LAZ ≥−2 but <−1). Pakistani children were enrolled based on weight-for-length Z score <−2. Zambian children were enrolled based on LAZ, weight-for-height Z score, or weight-for-age Z score <−2. The children enrolled in the studies were given various nutritional supplementation. Children whose wasting status did not improve with these nutritional supplementations, and for whom no medical condition was identified, were evaluated by a pediatric gastroenterologist to determine eligibility criteria for endoscopy. Cohorts from the North American participants were considered not to have EED and served as comparator groups; these children had *1*) clinical, histologic, and serologic manifestations of gluten-sensitive enteropathy (celiac disease) or *2*) no evidence of gluten-sensitive enteropathy and no pathologic abnormality (NPA) noted in the original pathology report. Hematoxylin and eosin (H&E)–stained histologic sections from the biopsies were scanned into digital slide format and subsequently scored for a panel of histologic parameters as described in Supplementary Table 2 and detailed in a companion paper [23].

### Tissue microarray construction

To increase the efficiency of subsequent analyses of histologic sections, formalin-fixed paraffin-embedded blocks were obtained from all centers except Cincinnati Children’s Hospital Medical Center and used to construct center-specific tissue microarrays (TMAs). H&E-stained slides of the tissue blocks were examined by a pathologist (CAM), and the most representative areas of tissue were circled with an indelible marker for inclusion in the TMAs. Cases were rejected if the remaining tissue in the paraffin blocks was not identified. For acceptable cases, 3-mm sampling core needles were used, which was generally sufficient to remove the entirety of a tissue biopsy fragment from the donor blocks and transfer it to the TMA block. If the study sites had separated the biopsies into separate blocks and designated one block as belonging to either the first part of the duodenum or the second or third segments, then the more distal tissue was used. If more than one tissue fragment was present in a donor block, a corresponding H&E-stained slide was evaluated by a pathologist (CAM) to identify the largest and/or most representative tissue fragment for transfer to the TMA block. The TMAs were constructed using a TMArrayer (Pathology Devices) in the Biorepository and Tissue Research Facility at the University of Virginia School of Medicine.

### Antibodies and IHC

**Figure 1:**
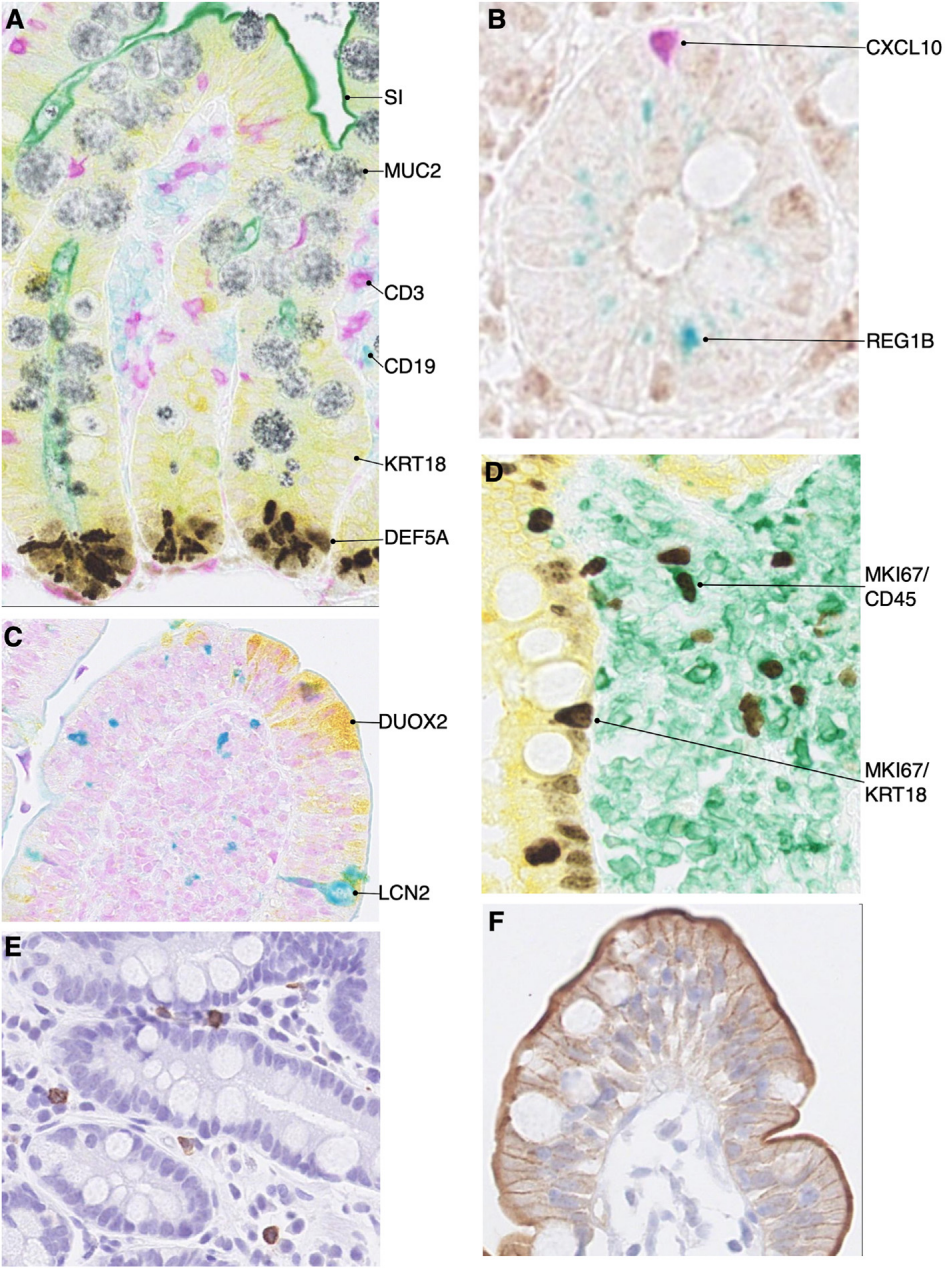
Examples of immunohistochemistry panel staining. Environmental enteric dysfunction (EED)1 immunohistochemistry (IHC) panel with SI (green), MUC2 (silver-black), CD3 (purple), CD19 (teal), KRT18 (yellow) and DEFA5 (brown) (A). EED2 IHC panel with CXCL10 (purple) and REG1B (green) (B). EED3 panel with DUOX2 (yellow) and LCN2 (teal) (C). EED4 IHC panel with KRT18 (yellow), CD45 (green) and MKI67 (brown) (D). For analysis, MKI67 was quantified separately when occurring in yellow zones (MKI67/KRT18) and green zones (MKI/CD45). Granzyme B (brown) (E). SLC15A1 (brown) (F). CD, cluster of differentiation; CXCL10, C-X-C motif chemokine 10; DEFA5, defensin alpha 5; DUOX2, dual oxidase 2; KRT18, cytokeratin 18; LCN2, lipocalin 2; MKI67, Antigen Kiel 67; MUC2, mucin 2; REG1B, regenerating family 1 beta; SI, sucrase isomaltase.

The antibodies selected for this study were chosen in an iterative process by a committee of the 3 coordinating investigators, each of the center principal investigators, and 2 of the study pathologists. Gene products associated with various aspects of immune cell and epithelial differentiation and function were chosen based on committee members’ assessment of their potential relevance to EED pathophysiology. Because of the small size of the tissues, markers sought were prioritized by consensus vote in tiers of interest, aiming for panel size of approximately 15–20 IHC stains. To limit the number of tissue sections used, whenever possible, antibodies were combined into multiplexed stains, with each probe visualized using a different colored chromogen in consecutive rounds of antibody binding and chromogen application on a robotic platform. The final antibody panels used were the result of an iterative process that included *1*) finding suitable antibody reagents and *2*) acceptable performance of antibodies during IHC titration and validation on control tissues (normal and inflamed samples of human small intestine). Details about the antibodies and staining procedure are found in the Supplementary Methods and Supplementary Table 3. Table 1 lists antigens detected by the IHC panels and corresponding chromogens, and Figure 1 portrays representative IHC stains with these panels. In deployment, the antibody for tight junction protein 1/zona occludens 1 produced diffuse nonspecific staining and was excluded from further analysis.

**Table 1:** Antigens targeted by immunohistochemistry.

Antigen	Gene symbol	IHC panel	Chromogen color	Expected tissue target/staining
CD3	*CD3*	EED1	Purple	T lymphocytes
CD19	*CD19*	EED1	Teal	B lymphocytes
Cytokeratin 18	*KRT18*	EED1	Yellow	All enterocyte cell cytoplasm
Defensin alpha 5	*DEFA5*	EED1	Brown	Paneth cells
Mucin 2	*MUC2*	EED1	Silver-black	Goblet cells
Sucrase isomaltase	*SI*	EED1	Green	Enterocyte brush border
C-X-C motif chemokine 10	*CXCL10*	EED2	Purple	Monocytic immune cells
Regenerating Family 1 beta	*REG1B*	EED2	Green	Cytoplasm of epithelial cell subsets
Tight junction protein 1/Zona occludens 1	*TJP1*/*ZO1*	EED2	Brown	Apical surfaces of enterocytes
Dual oxidase 2	*DUOX2*	EED3	Yellow	Cytoplasm of epithelial cell subsets
Lipocalin 2	*LCN2*	EED3	Teal	Subsets of epithelial cells and monocytes
Leucocyte common antigen	*CD45*	EED4	Green	All leukocytes
Ki-67	*MKI67*	EED4	Brown	All cells in S phase of cell cycle
Cytokeratin 18	*KRT18*	EED4	Yellow	All enterocyte cell cytoplasm
Granzyme B	*GZMB*	—	Brown	Lymphocyte subsets
Solute carrier family 15 member 1	*SLC15A1*	—	Brown	Enterocyte brush border

Abbreviations: EED, environmental enteric dysfunction; IHC, immunohistochemistry.

### Image analysis

The immunohistochemical sections were digitally scanned (NanoZoomer S360, Hamamatsu). Digital files were managed in the PathCore Flow software suite. TMA image files were dearrayed into individual image files for each tissue sample using the Visopharm ONCOTOPIX software suite, which was subsequently used for image analysis. Details of the image analysis algorithms can be found in the Supplementary Methods.

Because the biopsies varied in size, we normalized IHC quantification by tissue area; in most cases final data output corresponded to either the area of detected chromogen deposition or the number of segmented stained cells divided by the area of the tissue region of interest. Such data output is designated by the suffix “_SA”. For those markers that were detected only within epithelial cells, the total area of epithelium present in a biopsy, as detected by cytokeratin 18 staining, was used as the normalizer. Such data output is designated by the suffix “_EA”. To detect intraepithelial lymphoctes (IELs) algorithms were designed to measure the staining area (IELa) and cell count (IELc) for cluster of differentiation 3 (CD3) (a marker of T lymphocytes) only within areas of cytokeratin 18 staining.

### Statistical methods

To examine the relationships between IHC markers and disease, we modeled the IHC markers as outcome/dependent variables and disease states (EED compared with celiac and EED compared with NPA) as predictor/independent variables. We also modeled IHC markers as outcome/dependent variables and histopathology parameters as predictors/independent variables to examine their relationships among the biopsies from the cohorts. We used unrounded consensus histologic scores, calculated as averages across the reads of 2 or 3 Consortium pathologists for each parameter per slide image derived from histologic slides of the paraffin tissue blocks used for the IHC analysis [23]. The specific IHC marker–histopathology parameter relationships for testing were specified a priori, based on hypotheses about clinically plausible associations. We initially performed univariate linear regressions. Because of the exploratory nature of this study, univariate associations with a *P* value of <0.1 (2-tailed) were tested in subsequent multivariable models.

Multivariable models were adjusted for sex and 3 slide quality parameters: histologic quality, drying/crush artifact, and tissue orientation. Models assessing IHC markers and histologic parameters were also adjusted for age. Models of IHC markers’ relationships with disease status were not adjusted for age because of collinearity of age and disease, intrinsic to the study design; collinearity was evaluated by variance inflation factors and the age variable’s influence on SEs. NPA was the baseline group in IHC and disease models. For multivariable models, *P* value <0.05 (2-tailed) was considered statistically significant. Confidence intervals (CIs) from both univariate and multivariable analyses are presented as 95% intervals for comparability.

Because the residuals in models with untransformed IHC values were not normally distributed, IHC markers were log transformed. IHC values of 0 were replaced with the midpoint between the lowest value for that variable and 0. Coefficients on the log scale were exponentiated before interpretation; the exponentiated coefficient represents the percentage change in the dependent variable with 1 unit increase of the independent variable.

Analyses were conducted using R version 4.1.3 (The R Project for Statistical Computing, The Free Software Foundation).

## Results

### Study populations

The demographic features of the members of the cohorts who provided tissue for IHC analysis are presented in Table 2 and represent a subset of those described in the Environmental Enteric Dysfunction Biopsy Initiative Consortium companion paper [22] due to tissue availability (sufficient residual tissue in the paraffin blocks) (Supplementary Figure 1). Due to the small size of the biopsies, for some cases, insufficient tissue for evaluation was present in the histologic sections of the TMAs used for the immunohistochemical analysis. The percentage missing data for the variables analyzed ranged from 0 to 19.08% (Supplementary Table 4).

**Table 2:** Demographic characteristics of participants by site and disease status, median values (SD).

	BEED	SEEM	BEECH	EED sites combined	UVa Celiac	CCHMC Celiac	United States Celiac combined	UVa NPA^5^	CCHMC NPA^5^	United States NPA combined^5^
	*n* = 117	*n* = 60	*n* = 56	*n* = 233	*n* = 2	*n* = 10	*n* = 12	*n* = 16	*n* = 8	*n* = 24
Age^1^, y, mean (SD)	1.6 (0.2)	1.6 (0.3)	1.6 (0.3)	1.6 (0.2)	6.3 (5.1)	6.7 (2.2)	6.6 (2.5)	11.8 (4.1)	3.5 (1.0)	9.0 (5.2)
Age^1^, y, median [IQR]	1.6 [1.4, 1.7]	1.7 [1.3, 1.8]	1.6 [1.4, 1.8]	1.6 [1.4, 1.8]	6.3 [4.5, 8.1]	6.3 [5.2, 7.2]	6.3 [5.0, 7.9]	3.5 [2.8, 3.9]	12.1 [7.6, 15.6]	7.5 [4.1, 13.4]
Sex, %F	58.1%	31.6%	44.6%	49.4%	50%	50%	50%	50%	50%	50%
LAZ^2^, median [IQR]	−2.1 [−2.8, −1.5]	−3.2 [−3.7, −2,4]	−3.2 [−3.8, −2.8]	−2.7 [−3.4, −1.9]	0.6 [0.0, 1.1]	0.4 [−0.5, 1.1]	0.4 [−0.6, 1.2]	−0.3 [−1.1, 1.1]	0.2 [−0.2, 1.1]	0.2 [−0.9, 1.1]
WAZ^2^, median [IQR]	−1.7 [−2.3, −1.2]	−3.1 [−3.6, −2.6]	−2.2 [−2.7, −1.8]	−2.2 [−2.9, −1.6]	0.9 [0.1, 1.7]	0.2 [−0.3, 0.3]	0.2 [−0.4, 0.5]	0.7 [−0.5, 1.6]	−0.4 [−1.4, 0.6]	0.3 [−0.9, 1.4]
WHZ^2^^,^^3^, median [IQR]	−1.0 [−1.4, −0.4]	−2.2 [−2.7, −1.8]	−0.9 [−1.4, −0.2]	−1.2 [−1.9, −0.5]	−0.6 [−0.6, −0.6]	−0.7 [−0.8, −0.7]	−0.6 [−0.7, −0.6]	−0.5 [−0.9, −0.1]	−	−0.5 [−0.9, −0.1]
BMI^2^^,^^4^, median [IQR]	−	−	14.7 [14.6, 15.2]	14.7 [14.6, 15.2]	19.0 [17.0, 20.9]	14.9 [14.4, 15.5]	15.1 [14.5, 16.3]	14.9 [14.7, 15.2]	18.4 [16.2, 23.4]	16.5 [15.1, 20.9]

Abbreviations: BEECH, Biomarkers of Environmental Enteropathy in Children; BEED, Bangladesh Environmental Enteric Dysfunction; CCHMC, Cincinnati Children’s Hospital Medical Center; EED, environmental enteric dysfunction; F, female; LAZ, length-for-age Z score; NPA, no pathologic abnormality; WAZ, weight-for-age Z score; WHZ, weight-for-length Z score; SEEM, Study of Environmental Enteropathy and Malnutrition; UVa, University of Virginia.

1 At the time of biopsy.

2 Closest to the time of biopsy.

3 Weight-for-height Z score can only be calculated for children <5 y. All EED cohorts had children below 5 y; CCHMC Celiac had 2; UVa Celiac included 1, CCHMC Normal included 7 children.

4 BMI is used as a measure of thinness (or overweight status) among children >2 y. All United States cohorts had children >2 y. BEECH cohort included 7 children > 2 y.

5 Comparator cohort with NPA detected in duodenal biopsy histology.

### EED compared with NPA

**Figure 2:**
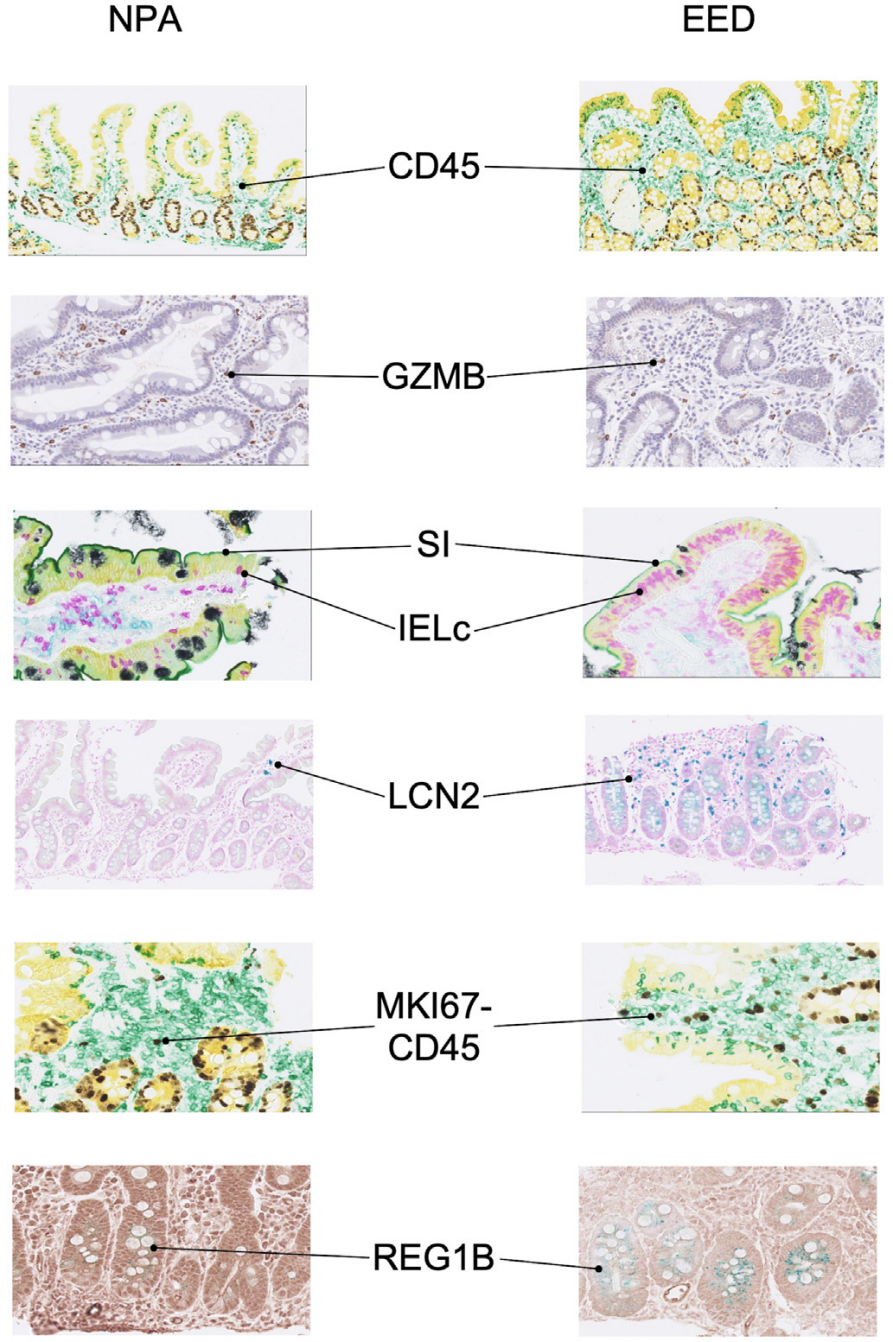
Examples of differential immunohistochemistry (IHC) staining in NPA and EED tissues. Left column: Images of NPA IHC-stained histologic sections. Middle column: List of antigens detected in IHC stains. Right column: Images of EED IHC-stained histologic sections. From top: CD45 is stained with green, GZMB is stained brown, SI is stained green, intraepithelial lymphocytes are identified by CD3 staining (purple) within the epithelial layer detected by cytokeratin 18 (yellow), LCN2 is stained with teal, MKI67 is stained brown and is identified within leukocytes by CD45 staining (green), REG1B is stained green. EED, environmental enteric dysfunction; GZMB, granzyme B; IELc, counts of intraepithelial lymphocytes; LCN2, lipocalin 2; NPA, no pathologic abnormality; REG1B, regenerating family 1 beta; SI, sucrase isomaltase.

In quantitative univariate analysis of immunohistochemical staining in the EED cohorts and that of American cohorts with NPA, 7 IHC parameters differed significantly (*P* < 0.1) in EED compared with NPA tissues, including elevated cluster of differentiation 45 (CD45) (a pan-marker of leukocytes), normalized to either tissue surface area (84%; 95% CI: 14%, 198%) or epithelial area (68%; 95% CI: 24%, 127%) (Table 3); lower expression of granzyme B (GZMB) (a product of cytotoxic T cells and natural killer [NK] cells); elevated expression of lipocalin 2 (LCN2) (a component of innate immunity) (309%; 95% CI: 77%, 843%); elevated expression of regenerating family 1 beta (REG1B) (an effector of cell regeneration and survival) (177%; 95% CI: 38%, 455%); and lower expression of sucrase isomaltase (SI) (−62%; 95% CI: −76%, −40%) and solute carrier family 15 member 1 (SLC15A1) (−37%; 95% CI: −61%, 1%) (both nutrient transporters expressed in enterocyte brush borders). Two additional parameters, measured as numerical counts, significantly differed between these 2 groups: counts of IELs (IELc) (31%; 95% CI: −2%, 73%) and Antigen Kiel 67 (MKI67) (a cell proliferation marker) in the leukocyte compartment (MKI67-CD45) (110%; 95% CI; 15%, 285%) were both elevated in EED. As shown in Table 3, in multivariable analysis, 7 of these parameters remained statistically different (*P* < 0.05 between the NPA and EED cohorts): CD45 (80%; 95% CI: 24%, 127%), GZMB (−74%; 95% CI: −82%, −62%), IELc (49%; 95% CI: 9%, 105%), LCN2 (659%; 95% CI: 198%, 1838%), MKI67-CD45 (267%; 95% CI: 92%, 601%), REG1B (221%; 95% CI: 47%, 600%), and SI (−58%; 95% CI: −75%, −29%). Figure 2 shows examples of the differences in staining of these markers in NPA and EED tissues.

**Table 3:** Comparison of immunohistochemistry parameters in environmental enteric dysfunction compared with no pathologic abnormality tissue samples.

IHC parameter	Univariate analysis			Multivariate analysis		
	Effect size^1^ (EED vs. NPA)	95% CI	*P*	Effect size^1^ (EED vs. NPA)	95% CI	*P*
CD19_EA	+10%	(−30%, 71%)	0.683	—	—	—
CD19_SA	+11%	(−21%, 55%)	0.555	—	—	—
CD3_EA	−11%	(−42%, 37%)	0.592	—	—	—
CD3_SA	−9%	(−36%, 29%)	0.59	—	—	—
CD45_EA	+68%	(24%, 127%)	<0.001	+80%	(28%, 153%)	<0.001
CD45_SA	+84%	(14%, 198%)	0.013	+57%	(−8%, 167%)	0.095
CXCL10_SA	+9%	(−48%, 130%)	0.811	—	—	—
DEFA5_EA	−22%	(−53%, 30%)	0.345	—	—	—
DUOX2_EA	−29%	(−88%, 327%)	0.708	—	—	—
GZMB_SA	−32%	(−55%, 3%)	0.068	−74%	(−82%, −62%)	<0.001
IELa_EA	+16%	(−21%, 69%)	0.453	—	—	—
IELc_EA	+31%	(−2%, 73%)	0.066	+49%	(9%, 105%)	0.014
LCN2_SA	+309%	(77%, 843%)	<0.001	+659%	(198%, 1838%)	<0.001
MKI67-K18_EA	+22%	(−45%, 17%)	0.163	—	—	—
MKI67-CD45_SA	+110%	(15%, 285%)	0.016	+267%	(92%, 601%)	<0.001
MUC2_EA	−20%	(−45%, 17%)	0.245	—	—	—
REG1B_EA	+177%	(38%, 455%)	0.004	+221%	(47%, 600%)	0.004
SI_EA	−62%	(−76%, −40%)	<0.001	−58%	(−75%, −29%)	0.001
SLC15A1_EA	−37%	(−61%, 1%)	0.055	−27%	(−57%, 25%)	0.253

Abbreviations: CD, cluster of differentiation; CI, confidence interval; CXCL10, C-X-C motif chemokine 10; DEFA5, defensin alpha 5; DUOX2, dual oxidase 2; EED, environmental enteric dysfunction; GZMB, granzyme B; IELa, intraepithelial lymphocyte surface area; IELc, counts of intraepithelial lymphocytes; IHC, immunohistochemistry; LCN2, lipocalin 2; KRT18, cytokeratin 18; MKI67, antigen Kiel 67; MUC2, mucin 2; NPA, no pathologic abnormality; REG1B, regenerating family 1 beta; SI, sucrase isomaltase; SLC15A1, solute carrier family 15 member 1.

1 Effect size represents the exponentiated coefficients, indicating the percent change in the dependent variable between EED and NPA.

### EED compared with celiac disease

As shown in Table 4, in the analysis of IHC staining in the EED and celiac disease cohorts, 8 IHC markers differed between the 2 groups at the *P* < 0.1 level. However, after adjusting for sex and technical slide quality parameters in multivariable analysis, only 2 markers significantly differed between these groups at the *P* < 0.05 level: both CD19 normalized to surface area (−43%; 95% CI: −66%, −4%) and CD3 normalized to surface area (−46%; 95% CI: −68%, −8%) were lowered in EED compared with celiac disease.

**Table 4:** Comparison of immunohistochemistry parameters in environmental enteric dysfunction compared with celiac disease tissue samples.

IHC parameter	Univariable analysis			Multivariable analysis		
	Effect size^1^ (EED vs. celiac)	95% CI	*P*	Effect size^1^ (EED vs. celiac)	95% CI	*P*
CD19_SA	−54%	(−71%, −26%)	0.001	−43%	(−66%, −4%)	0.036
CD19_EA	−59%	(−78%, −24%)	0.005	−44%	(−72%, 10%)	0.092
CD3_SA	−48%	(−68%, −16%)	0.008	−46%	(−68%, −8%)	0.024
CD3_EA	−55%	(−76%, −17%)	0.011	−47%	(−73%, 4%)	0.064
CD45_SA	−58%	(−91%, 104%)	0.279	—	—	—
CD45_EA	−47%	(−80%, 41%)	0.2	—	—	—
CXCL10_SA	−51%	(−96%, 489%)	0.569	—	—	—
DEFA5_EA	−12%	(−57%, 79%)	0.72	—	—	—
DUOX2_EA	+92%	(−84%, 2251%)	0.607	—	—	—
GZMB_SA	+34%	(−22%, 133%)	0.291	—	—	—
IELa_EA	−38%	(−64%, 5%)	0.077	−9%	(−49%, 60%)	0.737
IELc_EA	−17%	(−44%, 24%)	0.364	—	—	—
LCN2_SA	−76%	(−93%, −24%)	0.015	−52%	(−87%, 74%)	0.265
MKI67-K18_EA	−69%	(−90%, −6%)	0.038	−41%	(−80%, 72%)	0.332
MKI67-CD45_SA	+7%	(−84%, 634%)	0.946	—	—	—
MUC2_EA	+12%	(−34%, 90%)	0.671	—	—	—
REG1B_EA	+16%	(−89%, 1098%)	0.901	—	—	—
SI_EA	−1%	(−47%, 88%)	0.986	—	—	—
SLC15A1_EA	−83%	(−96%, −18%)	0.028	−78%	(−96%, 8%)	0.062

Abbreviations: CD, cluster of differentiation; CI, confidence interval; CXCL10, C-X-C motif chemokine 10; DEFA5, defensin alpha 5; DUOX2, dual oxidase 2; EED, environmental enteric dysfunction; GZMB, granzyme B; IELa, intraepithelial lymphocyte surface area; IELc, counts of intraepithelial lymphocytes; IHC, immunohistochemistry; LCN2, lipocalin 2; KRT18, cytokeratin 18; MKI67, antigen Kiel 67; MUC2, mucin 2; REG1B, regenerating family 1 beta; SI, sucrase isomaltase; SLC15A1, solute carrier family 15 member 1.

1 Effect size represents the exponentiated coefficients, indicating the percent change in the dependent variable between EED and celiac disease.

### Association of IHC parameters with histologic scoring

A subset of 8 digitally quantified IHC markers was predicted to mirror specific histologic parameters (H&E) scored by a team of pathologists examining sections from the same sets of tissue specimens. All 8 IHC parameters were significantly associated with histologic scoring parameters in univariate analyses (Table 5): CD45_SA (29%; 95% CI: 0, 65%) and CD19_SA (32%; 95% CI: 13%, 55%) were associated with chronic inflammation, IELa (35%; 95% CI: 22%, 51%) and IELc (21%; 95% CI: 12%, 32%) were associated with histologic scoring for IELs, CD45_EA (9%; 95% CI: 0, 18%) and CD19_EA (12%; 95% CI: 0, 25%) were associated with histologic scoring of abnormalities in villus architecture, defensin alpha 5 (a marker of Paneth cells) (−19%; 95% CI: −30%, −5%) was associated with histologic scoring of Paneth cells, and mucin 2 (a marker of goblet cells) (−15%; 95% CI: −25%, −4%) was associated with histologic scoring of goblet cells. As shown in Table 5, 4 of these associations remained significant in multivariable analysis: CD19_SA (33%; 95% CI: 13%, 55%) with chronic inflammation, both IELa (31%; 95% CI: 18%, 47%) and IELc (19%; 95% CI: 9%, 29%) with IELs, and CD19_EA (13%; 95% CI: 1%, 27%) with villus architecture.

**Table 5:** Comparison of selected immunohistochemistry parameters with histologic scores.

Histology parameter	IHC parameter	Univariable model			Multivariable model		
		Effect size^1^ (per unit of histologic score)	95% CI	*P*	Effect size^1^ (per unit of histologic score)	95% CI	*P*
Chronic inflammation	CD45_SA	29%	(0, 65%)	0.046	18%	(−8%, 50%)	0.197
Chronic inflammation	CD19_SA	32%	(13%, 55%)	<0.001	33%	(13%, 55%)	<0.001
Intraepithelial lymphocytes	IELa_EA	35%	(22%, 51%)	<0.001	31%	(18%, 47%)	<0.001
Intraepithelial lymphocytes	IELc_EA	21%	(12%, 32%)	<0.001	19%	(9%, 29%)	<0.001
Villus architecture	CD19_EA	12%	(0, 25%)	0.044	13%	(1%, 27%)	0.03
Villus architecture	CD45_EA	9%	(0, 18%)	0.047	7%	(−2%, 16%)	0.148
Paneth cell depletion	DEFA5_EA	−19%	(−30%, −5%)	0.008	−13%	(−29%, 6%)	0.166
Goblet cell depletion	MUC2_EA	−15%	(−25%, −4%)	0.009	−10%	(−22%, 3%)	0.130

Abbreviations: CD, cluster of differentiation; CI, confidence interval; DEFA5, defensin alpha 5; IELa, intraepithelial lymphocyte surface area; IELc, counts of intraepithelial lymphocytes; IHC, immunohistochemistry; MUC2, mucin 2.

1 Effect size represents the exponentiated coefficients, indicating the percent change in the dependent variable with 1 unit increase in histologic score.

## Discussion

IHC is a standard procedure that permits visualization of antigen expression in tissue sections to show the compartment in which the targets are expressed. The technique has been limited in the past by the number of antigens that can be analyzed in a single histologic section, but with the addition of new chromogens in recent years and technical advances in serial IHC, studies like ours are possible where up to 6 different antigens may be interrogated in a single histologic section. This capacity greatly extends our ability to study rare and limited tissue samples, as in the case of endoscopic biopsies from children with EED, and permits more analyses to be performed than with typical single-chromogen IHC, which rapidly exhausts small tissue blocks. Additionally, our approach uses a commercially available digital slide scanning platform and image analysis quantification software to provide greater precision and reproducibility than human interpretation of the immunohistochemical staining.

Our findings indicate that leukocyte infiltration, measured by CD45, and IELs, measured as CD3-positive cells within the epithelial layer marked by keratin 18 staining, are significantly elevated in duodenal tissue in children with EED relative to comparator children from North America without histologic evidence of any enteropathy. These findings are similar to the well-documented histologic changes previously described as tropical enteropathy [3] or environmental enteropathy [4,5] and were also identified in the companion histologic analysis of this EED cohort [23]. A novel finding of this study is the significantly elevated leukocyte proliferation in EED, as evidenced by MKI67 expression in CD45-positive cells, suggesting that the elevated immune cell infiltrate in EED is due in part to local proliferation of immune cells, most likely in response to ongoing antigen-driven stimulation. Although our study did not specifically address which immune cell compartment(s) were most involved in this proliferative drive, the technique we used could easily be adapted in future studies by costaining MKI67 with markers of subsets of immune cells.

Our data also indicate that aspects of the innate immune response are upregulated in EED, including LCN2. Visual inspection of the stains shows that this elevated expression occurred in both epithelial and immune cell compartments in EED (Figure 2). LCN2 is a secreted protein that binds small hydrophobic molecules and plays a role in many physiologic and pathologic processes [24]. Its role in innate immunity is thought to be the sequestration of iron, limiting bacterial growth [25], and to ameliorate deleterious stimuli in the gut to promote homeostasis of gut microflora [26]. Previous transcriptomic studies also found that LCN2 is upregulated in gut tissue in EED [21,27]; our study extends this finding to a larger cohort of EED cases and shows that both epithelial and immune cell expression contribute to the higher levels of expression seen in EED.

We also found REG1B to be upregulated in EED, with signal located to the lower intestinal crypt epithelium, which corroborates RNA in situ hybridization studies of *REG1B* gene expression [28]. REG1B is a multifunctional protein that serves a proproliferative role in intestinal epithelium and is also antiapoptotic and essential for the generation and maintenance of the crypt–villus growth axis of the small intestinal mucosa [29]. In a murine model of amoebiasis, REG1B was found to upregulated in colonic mucosa and to protect cells from infection-induced apoptosis [30]. IHC signals of LCN2 and REG1B in our study indicate that gut epithelial cells in EED sense stimuli similar to those seen in infectious processes, lending further support that EED may in part be related to epithelial stress because of chronic exposure to pathogens and/or altered gut microbial community structure. REG1B protein has been previously shown to be elevated in the stool of children with EED, and levels prospectively predicted future growth shortfalls [31].

Our study also demonstrated that enterocyte brush border transporter proteins are significantly lowered in EED. Although SLC15A1 only showed a significantly lower level in univariate analysis, the SI protein was significantly lowered in EED in multivariable analysis. Visual inspection of both SI and SLC15A1 stains showed a significantly lower level in EED specific to the functional location on the epithelial brush border (Figure 2). These results extend a previous EED transcriptomic analysis, where it was found that many transporter genes were downregulated in the duodenum, including SLC15A1 [20]. Maintenance of the brush border is one defining element of the most mature pattern of differentiation of enterocytes and is critical to the ability of the gut to provide the level of nutrient absorption required to maintain a robust nutrient state. Our IHC analysis is consistent with the recently postulated model [32] of the gut epithelium in EED responding to chronic stress signals, leading to a decreased differentiation program that culminates in decreased capacity to absorb nutrients, leading to malnutrition and stunting.

One surprising finding was the significant loss of GZMB protein in EED tissue samples. GZMB is a serine protease expressed by cytotoxic T lymphocytes and NK cells. Though best known for perforin-dependent apoptotic killing of cells, GZMB also participates in other functions such as cleaving of extracellular matrix components, cytokines, cell receptors, and angiogenic and clotting proteins [33]. The results are unexpected since in the companion gene expression study of this cohort, the RNA transcript of *GZMB* was markedly upregulated in biopsies of duodenal epithelium [34]. IHC generally cannot identify proteins that have been secreted outside of cells and are present in extracellular fluid because the concentration of the diffused protein is beneath the level of detection. We postulate that the cytotoxic T lymphocytes and NK cells of the gut mucosa are actively stimulated to secrete GZMB and that the upregulation of gene transcript cannot replenish the normal intracellular stores of GZMB. If true, this may be consistent with the “lymphocyte exhaustion” concept, as noted in chronic viral infection and tumor immunology [35,36]. Lymphocyte exhaustion is an immunosuppressive state characterized by alterations in effector lymphocytes leading to a decreased ability to fight infection. Supporting this hypothesis is the observation that *GZMB* mRNA transcript upregulation, as seen in EED, is associated with T cell exhaustion [35,37]. The role of lymphocyte exhaustion in EED is worthy of further pursuit as a factor in the chronic inflammatory state that underlies this disorder and its association with higher rates of live oral vaccine failure in low-income countries [38].

It has long been known that the histologic changes in EED resemble those in celiac disease, with altered (blunted) villus architecture and elevated chronic inflammatory infiltrate of the lamina propria and IELs. Given the well-understood genetic and immunologic parameters that underlie celiac disease [39], there is the continued impression that comparison of these 2 disease entities may shed light on pathophysiologic similarities and differences that underlie EED. Our IHC panel confirms similarity between these 2 entities, as we demonstrated only a few meaningful differences, with only the predominance of CD19 (B lymphocytes) and CD3 (T lymphocytes) being significantly higher in celiac disease relative to the EED samples. This corroborates histologic analyses presented in a companion paper [23] in which the inflammatory changes in EED were less pronounced than in celiac disease.

Several IHC parameters were associated with specific histologic parameters from the same specimens, as scored by a study group of pathologists, using a histologic index created for EED [23,40]. This index has been proposed for use in identifying EED severity and evaluating response to therapeutic interventions where duodenal biopsies are available. Although several IHC parameters show statistical association with cognate histologic scoring data association, small coefficients indicate significant variation between histologic scoring and IHC data for corresponding biopsies. One explanation is that while all available tissue was used for histologic scoring in a biopsy, the IHC stains were performed on TMAs that may have contained a subset of the tissue available in the original biopsy tissue block. Although an objective machine-standardized approach to histologic tissue assessment has great appeal and warrants further investigation, additional comparison studies are needed before an automated IHC readout can be recommended to supplant pathologist-determined scoring of H&E slides.

We acknowledge a limitation caused by the difficulty recruiting comparison cohorts locally or of similar age in North America because of ethical considerations in obtaining endoscopic biopsies from healthy children in LMICs and the relative infrequency of children undergoing endoscopy in the age range investigated in the EED cohorts, respectively. This resulted in a significant difference in age between the EED and comparator cohorts, and because age is collinear with disease status, we could not adjust for age in multivariable analyses of such comparisons without impairing the accuracy of the models’ estimates. Although the panel of IHC markers in this study is limited in the number of loci examined relative to the transcriptomic analyses recently performed on EED samples [[19], [20], [21]], the ability of IHC to identify the cell types and patterns of change of gene expression in complex tissue architecture lends additional information for understanding EED pathophysiology. Despite the limitations of this study, this is the largest IHC interrogation of EED duodenal samples performed to date and leverages a novel multiplexed IHC platform and objective digital quantification algorithms. Multiantibody panels and multiple chromogens are powerful tools to illuminate gut pathophysiology in a highly consequential disorder in children in LMICs worldwide.

## Acknowledgments

We thank Ms. Angela Miller in the Biorepository and Tissue Research Facility of the University of Virginia School of Medicine for assistance in the creation of tissue microarrays and subsequent histologic sectioning, Ms. Louise Warren of the University of Washington for assistance in manuscript preparation and Dr. Barbra Richardson of the University of Washington for statistical advice.

## Author contributions

The authors’ responsibilities were as follows – CAM, SRM, WAP, TK, TA, MMahfuz, SAA, PK: designed research; CAM, EB, SMKNB, ZJ, SRM, WAP, SR, TK, TA, MMahfuz, SAA, PK: conducted research; KV, SM, MMweetwa: analyzed data; CAM: wrote the paper; CAM: had primary responsibility for final content; CAM, TK, DMD, PBS, PIT: data management; CAM, DMD, KV, PBS, PIT: data interpretation; CAM, SR: generated histology score; and all authors: read and approved the final manuscript.

## Conflict of interest

The authors report no conflicts of interest.

## Funding

The Environmental Enteric Dysfunction Biopsy Initiative Consortium was funded by the following grants: Bill and Melinda Gates Foundation OPP1152812, OPP1066118, OPP1136759, OPP1138727, and OPP1144149, and Advanced Imaging and Tissue Analysis Core of the Washington University Digestive Diseases Research Core Center P30DK052574.

## Data availability

Data described in the manuscript, code book, and analytic code will be made available upon request to the corresponding author pending application and approval.

## Figures and Tables

**Table:** 

Surname	Name	Full Affiliation	Email
Ahmed	Kumail	Department of Paediatrics and Child Health, Aga Khan University, Karachi, Pakistan	kumail.ahmed@aku.edu
Ahmed	Sheraz	Department of Paediatrics and Child Health, Aga Khan University, Karachi, Pakistan	sheraz.ahmed@aku.edu
Alam	Md. Ashraful	Nutrition Research Division, International Centre for Diarrhoeal Disease Research, Bangladesh, Dhaka, Bangladesh	mashraful@icddrb.org
Das	Subhasish	Nutrition Research Division, International Centre for Diarrhoeal Disease Research, Bangladesh, Dhaka, Bangladesh	subhasish.das@icddrb.org
Denson	Lee A.	Division of Pediatric Gastroenterology, Hepatology, and Nutrition, Cincinnati Children's Hospital Medical Center, Cincinnati, OH, USA	Lee.Denson@cchmc.org
Fahim	Shah Mohammad	Nutrition Research Division, International Centre for Diarrhoeal Disease Research, Bangladesh, Dhaka, Bangladesh	mohammad.fahim@icddrb.org
Gazi	Md. Amran	Nutrition Research Division, International Centre for Diarrhoeal Disease Research, Bangladesh, Dhaka, Bangladesh	amran.gazi@icddrb.org
Haberman	Yael	Division of Pediatric Gastroenterology, Hepatology, and Nutrition, Cincinnati Children’s Hospital Medical Center, Cincinnati, OH, USA	Yael.Haberman@cchmc.org
Hasan	Md. Mehedi	Nutrition Research Division, International Centre for Diarrhoeal Disease Research, Bangladesh, Dhaka, Bangladesh	md.hasan@icddrb.org
Hossain	Md. Shabab	Nutrition Research Division, International Centre for Diarrhoeal Disease Research, Bangladesh, Dhaka, Bangladesh	dr.shabab@icddrb.org
Hotwani	Aneeta	Department of Paediatrics and Child Health, Aga Khan University, Karachi, Pakistan	aneeta.hotwani@aku.edu
Iqbal	Junaid	Department of Paediatrics and Child Health, Aga Khan University, Karachi, Pakistan	junaid.iqbal@aku.edu
Iqbal	Najeeha Talat	Department of Paediatrics and Child Health, Aga Khan University, Karachi, Pakistan	najeeha.iqbal@aku.edu
Jakhro	Sadaf	Department of Paediatrics and Child Health, Aga Khan University, Karachi, Pakistan	sadaf.jakhro@aku.edu
Kabir	Furqan	Department of Paediatrics and Child Health, Aga Khan University, Karachi, Pakistan	furqan.kabir@aku.edu
Liu	Ta-Chiang	Department of Pathology and Immunology, Washington University School of Medicine, St. Louis, MO, USA	ta-chiang.liu@wustl.edu
Mann	Barbara J.	Department of Medicine, University of Virginia School of Medicine, Charlottesville, VA, USA	bjm2r@virginia.edu
Marie	Chelsea	Department of Medicine, University of Virginia School of Medicine, Charlottesville, VA, USA	csm8r@virginia.edu
Mazumder	Ramendra Nath	Nutrition Research Division, International Centre for Diarrhoeal Disease Research, Bangladesh, Dhaka, Bangladesh	ramen@icddrb.org
Mudenda	Victor	Tropical Gastroenterology & Nutrition group, University of Zambia School of Medicine, Lusaka, Zambia	mudenda2003@yahoo.co.uk
Mulenga	Chola	Tropical Gastroenterology & Nutrition group, University of Zambia School of Medicine, Lusaka, Zambia	cholamulenga94@gmail.com
Qureshi	Abdul Khalique	Department of Paediatrics and Child Health, Aga Khan University, Karachi, Pakistan	abdul.qureshi@aku.edu
Rahman	Masudur	Department of Gastroenterology, Sheikh Russel National Gastroliver Institute and Hospital, Dhaka, Bangladesh	drmasud47@yahoo.com
Rahman	Najeeb	Department of Paediatrics and Child Health, Aga Khan University, Karachi, Pakistan	najeeb.rahman@aku.edu
Sadiq	Kamran	Department of Paediatrics and Child Health, Aga Khan University, Karachi, Pakistan	kamran.sadiq@aku.edu
Tearney	Guillermo J	Department of Pathology, Harvard Medical School, Boston, MA, USA	gtearney@partners.org
Umrani	Fayaz	Department of Paediatrics and Child Health, Aga Khan University, Karachi, Pakistan	fayaz.umrani@aku.edu
Yilmaz	Omer H	Department of Pathology, Massachusetts General Hospital, Boston, MA, USA	oyilmaz@mgh.harvard.edu
