# Supplementary material for: Multiplexed immunohistochemical evaluation of small bowel inflammatory and epithelial parameters in environmental enteric dysfunction
Source: Am J Clin Nutr. 2024 Sep 17;120(Suppl 1):S31–40. doi: 10.1016/j.ajcnut.2024.02.033 (PMC13169041; doi:10.1016/j.ajcnut.2024.02.033)
Supplement: Multimedia component 1 [file mmc1.zip › ajcnut_475_KELLEY~1_mmc1.DOC]

Supplementary Materials

Multiplexed immunohistochemical evaluation of small bowel inflammatory and epithelial parameters in environmental enteric dysfunction.

Kelley VanBuskirk, et al.


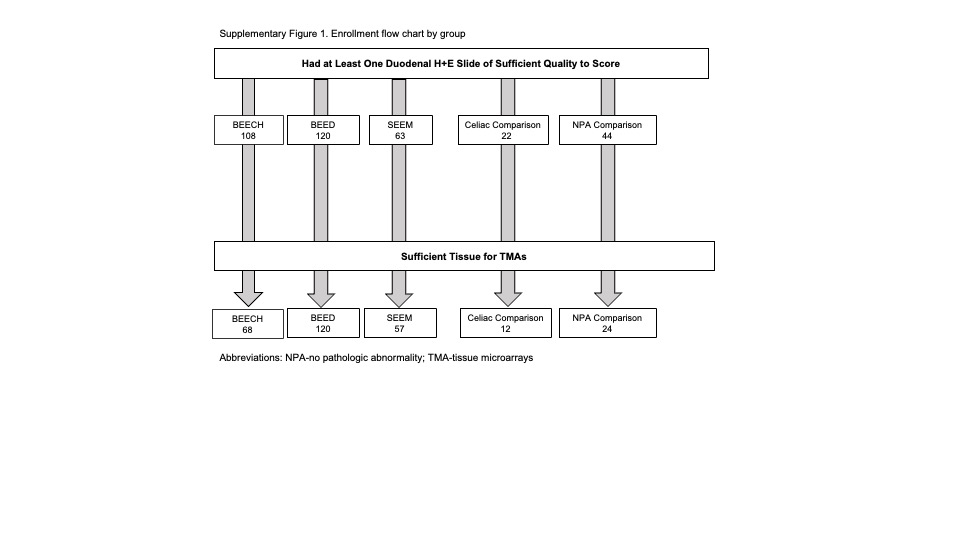


Supplementary Methods

Immunohistochemistry

All IHC stains, except for Granzyme B (GZMB), were performed on the Ventana Discovery Ultra (Roche Diagnostics) and IHC reagents and chromogens were from Roche Diagnostics. Antigen retrieval on this platform was performed using Cell Conditioner 1 (CC1) (64 minutes, 70°C). Chromogen deposition was mediated by horseradish peroxidase, which was deactivated by heat treatment between successive rounds of immunohistochemical staining in multiplexed assays. IHC for GZMB was performed on a Dako Autostainer after antigen retrieval using low pH Envision Flex Target Retrieval Solution (Dako) (20 minutes, 97°C). Supplementary Table 2 provides antibody sources and IHC conditions. In deployment, the antibody for Tight Junction Protein 1 / Zona Occludens 1 (TJP1/ZO1) resulted in diffuse non-specific staining and was excluded from further analysis.

Image analysis

The decision forest classification model in this software suite was trained to identify areas of tissue within each histologic image at 10x magnification, with a decision forest complexity setting of 20 (range 1-100) to define the region of interest (ROI). Subsequent similar classification models to define areas of specific chromogen deposition within the ROI were performed at 400x magnification with a decision forest complexity setting of 100. Output inspection showed that small areas of color detection (<5 microns) appeared to be artifacts and were gated out of the final output. The final output corresponded to the area of detected chromogen deposition. For certain stains, the segmentation algorithms within ONCOTOPIX were used to count individually stained cells within the ROIs. Three specific algorithms were developed to identify staining within the context of specific tissue components: 1)To detect intraepithelial lymphocytes (IELs), algorithms were designed to determine staining area and cell count for CD3 only within areas stained for keratin 18 (KRT18) (designated IELa and IELc, respectively); 2) to detect epithelial cells in S phase, algorithms were designed to determine staining area and cell count for MKI67 only within areas stained for KRT18 (designated MKI67-KRT18); 3) to detect leucocytes in S phase algorithms were designed to determine staining area and cell count for MKI67 only within areas stained for CD45 (designated MKI67-CD45).

Supplementary Table 1: Enrollment procedures by center

| Center | Enrollment period  Recruitment location | Recruitment strategy & eligibility criteria for nutritional intervention | Exclusion criteria for nutrition intervention | Nutritional intervention & non-response definition | Medical evaluation if failed nutritional intervention to exclude other causes of growth failure | Endoscopy timeframe |
| --- | --- | --- | --- | --- | --- | --- |
| BEECH, Zambia | October 1 2016 – May 31 2018  Urban slum in Misisi, Kuku, Chawama, and John Laing areas of Lusaka, Zambia | Door-to-door screening  1-18 month-olds with WAZ <-1 invited for clinic-based extended anthropometry; eligible if WAZ, LAZ, or WLZ <-2 | Caregiver unwilling for child to undergo HIV test and receive HIV care (if relevant), participating in another research study | Counseling on nutrition, water/sanitation/hygiene, and home care of child illness. Breastfeeding support and education. Starting at age 6 months, high energy protein supplement (corn-soya blend), 14 Eggs and 14 sachets of micronutrient powder provided every two weeks. Children with complicated SAM received hospital management and those with uncomplicated SAM were managed with ready-to-use therapeutic feeds on an outpatient basis per national protocols.  Non-response: LAZ or WLZ consistently <-2 after 3-4 months of nutritional supplementation | Examination by a pediatric gastroenterologist. Not eligible if chronic health condition that could cause growth faltering (e.g., cardiac disease), elevated tissue transglutaminase immunoglobulin A suggestive of celiac disease, current history of diarrhea, currently on antibiotics, hemoglobin <9g/dl, leukocytosis, elevated PT INR. | February 2, 2017 - June 7, 2019 |
| BEED, Bangladesh | July 17, 2016 - May 31, 2019  Urban slum in Baunibadh and Mirpur areas of Dhaka, Bangladesh | Door-to-door screening  12-18 month-olds with LAZ <-1 | - Severe acute malnutrition - History of persistent diarrhea - Known allergy to eggs or milk or milk intolerance | Directly observed on-site feeding of boiled egg and 150 ml whole milk at a study nutrition center 6 days/week for 90 days. Also received anti-helminthic treatment per national guideline, micronutrient sprinkles (one sachet daily for two months), and nutritional counseling for caregivers.  Non-response: For children with starting LAZ<-2, LAZ remains <-2. For children with starting LAZ <-1 but >-2, LAZ remains <-1. | Examination by a study physician. Ineligible if chronic health condition identified that could cause growth faltering (e.g., tuberculosis), elevated tissue transglutaminase immunoglobulin A suggestive of celiac disease, hemoglobin <8 g/dl^1^. Prior to endoscopy, examination and review of pre-anesthesia labs (clotting time, bleeding time, and PT INR) by a pediatric anesthetist. Ineligible if pre-anesthesia labs were abnormal^1^. If acute diarrhea, endoscopy deferred until resolved. | November 2, 2016 - August 26, 2019 |
| SEEM, Pakistan | March 1 2016 – November 2017  Matiari - a rural area 185 km from Karachi, Pakistan | Door-to-door screening  Eligible for monitoring if 3-6 months old and WLZ<-2. Nutritional counseling provided. If WLZ still <-2 at 9 months, eligible for nutrition intervention. | WLZ>0 and LAZ not <-1 on two consecutive visits. | Nutritional counseling for caregivers. Starting at age 9 months, AchaMuM - a ready-to-use supplementary food - 1 sachet daily for 2 months for WLZ<-2 but >-3. For WLZ<-3, weight-based supply was provided.   Non-response: No improvement in height and weight of child compared to preceding weight and height | Examination by two pediatricians after the completion of nutrition intervention. Ineligible if >24 months of age or if chronic health condition identified that could cause growth faltering (e.g., neurologic or cardiac disorders, tuberculosis). Four children had elevated serum levels of tissue transglutaminase immunoglobulin A. Repeat concentrations were normal while consuming a local traditional diet, which typically contains gluten; data from these children were retained in the SEEM cohort and Consortium analyses. Ineligible if hemoglobin <8g/dl, elevated PT INR^2^, or thrombocytopenia. | January 11, 2017 - October 10, 2018 |
| CCHMC, US | March 24, 2017 - March 5, 2019  Cincinnati Children’s Hospital Medical Center  Cincinnati, Ohio | Patients <12 years of age presenting for endoscopic intestinal biopsy for diagnostic purposes.^3^ Included two cohorts: 1) newly diagnosed celiac disease and 2) nondiagnostic group who had no medical diagnoses or diagnostic histology consistent with esophagogastrointestinal disease^4^ | N/A | N/A | N/A | March 24, 2017 - March 5, 2019 |
| UVa, US | June 7, 2017 - September 13, 2019  University of Virginia University Hospital Charlottesville, Virginia | Patients 1 - 18 years presenting for endoscopic intestinal biopsy for diagnostic purposes^3^ Included two cohorts: 1) newly diagnosed celiac disease and 2) nondiagnostic group who had no medical diagnoses or diagnostic histology consistent with esophagogastrointestinal disease^5^ | N/A | N/A | N/A | June 7, 2017 - September 13, 2019 |

## Supplementary Table 2: Histopathology scores of participants by site and disease status, median (interquartile range)

|  | BEED  N=117 | SEEM  N=60 | BEECH  N=56 | EED sites combined  N=233 | UVa Celiac  N=2 | CCHMC Celiac  N=10 | US Celiac combined  N=12 | UVa NPA^1^  N=16 | CCHMC NPA^1^  N=8 | US NPA combined^1^  N=24 |
| --- | --- | --- | --- | --- | --- | --- | --- | --- | --- | --- |
| Total Score Percent-5  (0-100%)  NS% | 58.3 (47.2, 66.7)  33.3% | 42.9 (35.7, 53.3)  11.7% | 50.0 (42.6, 58.3)  7.1% | 50.0  (41.7, 60.9)  21.5% | 55.5 (52.8, 58.3)  0% | 43.0 (31.3, 49.3)  0% | 47.2  (32.6, 50.7)  0% | 13.9 (7.6, 21.2)  0% | 16.4  (8.0, 22.2)  0% | 15.6  (7.6, 21.5)  0% |
| Goblet Cell Depletion  (0-4) / NS% | 2.0  (1.3, 2.5)  0% | 1.0  (0.5, 1.5)  0% | 1.5  (1.0, 2.0)  1.8% | 1.5  (1.0, 2.0)  0.4% | 0.4  (0.3, 0.4)  0% | 0.5  (0.5, 0.9)  0% | 0.5  (0.4, 0.8)  0% | 0.3  (0.0, 0.6)  0% | 0.0  (0.0, 0.1)  0% | 0.1  (0.0, 0.5)  0% |
| Intramucosal Brunner Glands  (0-3) / NS% | 0.0  (0.0, 0.5)  3.4% | 0.0  (0.0, 0.4)  1.7% | 0.0  (0.0, 0.5)  1.8% | 0.0  (0.0, 0.5)  2.6% | 1.0  (0.5, 1.5)  0% | 3.0  (3.0, 3.0)  0% | 3.0  (2.8, 3.0)  0% | 3.0  (1.2, 3.0)  0% | 0.8  (0.6, 2.6)  0% | 3.0  (0.9, 3.0)  0% |
| Intra-epithelial Lymphocytes  (0-4) / NS% | 1.5  (1.0, 2.0)  0.9% | 1.5  (1.0, 2.5)  0% | 1.0  (0.5, 1.5)  3.6% | 1.5  (1.0, 2.0)  1.3% | 2.5  (2.2, 2.8)  0% | 3.0  (2.5, 3.4)  0% | 3.0  (2.4, 3.1)  0% | 0.3  (0.0, 0.5)  0% | 0.4  (0.2, 0.8)  0% | 0.3  (0.0, 0.5)  0% |
| Paneth Cell Depletion  (0-3) / NS% | 3.0  (1.6, 3.0)  47% | 0.5  (0.3, 1.0)  16.7% | 1.5  (1.0, 2.5)  26.8% | 1.0  (0.5, 3.0)  34.3% | 1.1  (0.9, 1.3)  0% | 0.5  (0.3, 0.9)  0% | 0.5  (0.4, 1.1)  0% | 0.3  (0.2, 0.6)  0% | 0.0  (0.0, 0.5)  0% | 0.3  (0.0, 0.5)  0% |
| Villus Architecture  (0-4) / NS% | 2.0  (1.0, 3.0)  39.3% | 2.0  (1.0, 3.0)  28.3% | 2.0  (1.4, 4.0)  14.3% | 2.0  (1.0, 3.0)  30.5% | 4.0  (4.0, 4.0)  0% | 3.2  (1.8, 4.0)  0% | 4.0  (1.9, 4.0)  0% | 0.5  (0.3, 0.7)  18.8% | 0.2  (0.0, 0.5)  0% | 0.5  (0.0, 0.5)  12.5% |
| Chronic Inflammation  (0-3) / NS% | 1.5  (1.0, 1.5)  1.7% | 1.5  (1.0, 1.5)  1.7% | 1.5  (1.0, 2.0)  1.8% | 1.5  (1.0, 1.5)  1.7% | 2.2  (2.1, 2.4)  0% | 1.9  (1.5, 2.0)  0% | 2.0  (1.5, 2.1)  0% | 1.0  (0.6, 1.0)  0% | 0.5  (0.3, 1.0)  0% | 1.0  (0.5, 1.0)  0% |
| Enterocyte Injury  (0-3) / NS% | 0.3  (0.0, 0.5)  0% | 0.5  (0.0, 0.5)  0% | 0.0  (0.0, 0.5)  1.8% | 0.3  (0.0, 0.5)  0.4% | 1.1  (1.1, 1.2)  0% | 0.8  (0.5, 1.5)  0% | 1.0  (0.5, 1.5)  0% | 0.3  (0.0, 0.3)  0% | 0.0  (0.0, 0.1)  0% | 0.0  (0.0, 0.3)  0% |
| Epithelial Detachment  (0-4) / NS% | 1.0  (0.5, 1.0)  0% | 1.0  (0.7, 1.1)  0% | 1.0  (0.5, 1.0)  0% | 1.0  (0.5, 1.0)  0% | 0.8  (0.6, 0.9)  0% | 1.0  (1.0, 1.0)  0% | 1.0  (0.9, 1.0)  0% | 1.0  (1.0, 1.6)  0% | 0.8  (0.7, 1.0)  0% | 1.0  (0.7, 1.1)  0% |

^1^Comparator cohort with no pathologic abnormality (NPA) detected in duodenal biopsy histology

For each histology parameter and the summative Total Score Percent 5, the possible range of scores is provided in the first column. The NS% represents the proportion of slide images that were determined to be not scorable due to technical slide quality issues by the scoring pathologists.

Abbreviations: NPA, no pathologic abnormality; NS, Not Scorable

Supplementary Table 3: Antibodies and chromogens used in IHC

| Antigen gene symbol | Antibody source | Cat. # | Antibody type | Clone designation | Dilution | Chromogen |
| --- | --- | --- | --- | --- | --- | --- |
| CD19 | Abcam | ab134114 | Rabbit monoclonal | EPR5906 | 1:200 | DISCOVERY teal |
| CD3 | Dako | A0452 | Rabbit polyclonal | NA | 1:200 | DISCOVERY purple |
| CD45 | Dako | M0701 | Mouse monoclonal | 2B11+PD7/26 | 1:200 | DISCOVERY green |
| CXCL10 | Santa Cruz | sc-101500 | Mouse monoclonal | IP-10 | 1:100 | DISCOVERY purple |
| DEFA5 | Novus | NB110-60002 | Mouse monoclonal | 8C8 | 1:2000 | 3,3’-diaminobenzidine tetrahydrochloride (DAB). |
| DUOX2 | Millipore-Sigma | MABN787 | Mouse monoclonal | Duox S-12 | 1:50 | DISCOVERY yellow |
| GZMB | Abcam | ab4059 | Rabbit polyclonal | NA | 1:200 | 3,3’-diaminobenzidine tetrahydrochloride (DAB). |
| K18 | Abcam | ab32118 | Rabbit monoclonal | E431-1 | 1:50 | DISCOVERY yellow |
| LCN2 | Millipore-Sigma | HPA002695 | Rabbit polyclonal | NA | 1:2000 | DISCOVERY teal |
| MKI67 | Epitomics | ab1667 | Rabbit monoclonal | SP6 | 1:400 | 3,3’-diaminobenzidine tetrahydrochloride (DAB). |
| MUC2 | Abcam | Ab134119 | Rabbit monoclonal | EPR6145 | 1:1600 | DISCOVERY silver |
| REG1B | Sino Biological | 11638-R010 | Rabbit monoclonal | 010 | 1:100 | DISCOVERY green |
| SI | Novus | NBP1-87581 | Rabbit polyclonal | NA | 1:1600 | DISCOVERY green |
| SLC15A1 | Sino Biological | 203418-T10 | Rabbit polyclonal | NA | 1:200 | 3,3’-diaminobenzidine tetrahydrochloride (DAB). |
| TJP1 | ThermoFisher | 61-7300 | Rabbit polyclonal | NA | 1:600 | 3,3’-diaminobenzidine tetrahydrochloride (DAB). |

Supplementary Table 4: Missing IHC data

| Variable | Site or Study | | | | | Total missing data | % missing data |
| --- | --- | --- | --- | --- | --- | --- | --- |
|  | CCHMC | SEEM | BEECH | BEED | UVa |  |  |
| CD19_EA | 0 | 0 | 1 | 2 | 2 | 5 | 1.45% |
| CD19_SA | 0 | 0 | 1 | 1 | 1 | 3 | 0.87% |
| CD3_EA | 0 | 0 | 1 | 2 | 1 | 4 | 1.16% |
| CD3_SA | 0 | 0 | 1 | 2 | 1 | 4 | 1.16% |
| CD45_Ea | 0 | 0 | 1 | 2 | 2 | 5 | 1.45% |
| CD45_SA | 0 | 0 | 0 | 0 | 0 | 0 | 0.00% |
| CXLC10_SA | 0 | 0 | 0 | 0 | 0 | 0 | 0.00% |
| DEFA5_EA | 0 | 1 | 7 | 5 | 3 | 16 | 4.62% |
| DUOX2_EA | 0 | 4 | 28 | 12 | 8 | 52 | 15.03% |
| GZMB_SA | 0 | 3 | 29 | 12 | 11 | 55 | 15.90% |
| IELa_EA | 0 | 0 | 8 | 3 | 4 | 15 | 4.34% |
| IELc_EA | 0 | 0 | 8 | 3 | 4 | 15 | 4.34% |
| LCN2_SA | 0 | 4 | 28 | 12 | 8 | 52 | 15.03% |
| MKI67-CD45_SA | 0 | 6 | 29 | 12 | 12 | 59 | 17.05% |
| MKI67-K18_EA | 0 | 6 | 29 | 12 | 12 | 59 | 17.05% |
| MUC2_EA | 0 | 0 | 1 | 2 | 1 | 4 | 1.16% |
| REG1B_EA | 0 | 0 | 0 | 0 | 0 | 0 | 0.00% |
| SI_EA | 0 | 0 | 1 | 1 | 0 | 2 | 0.58% |
| SLC15A1_EA | 0 | 6 | 31 | 15 | 14 | 66 | 19.08% |
