# Supplementary material for: Multiplexed immunohistochemical evaluation of small bowel inflammatory and epithelial parameters in environmental enteric dysfunction
Source: Am J Clin Nutr. 2024 Sep 17;120(Suppl 1):S31–40. doi: 10.1016/j.ajcnut.2024.02.033 (PMC13169041; doi:10.1016/j.ajcnut.2024.02.033)
Supplement: Multimedia component 2 [file mmc2.zip › ajcnut_475_APPEND~1_mmc2.DOC]

Appendix A: The collaborators of the EEDBI Consortium

| **Surname** | **Name** | **Full Affiliation** | **Email** |
| --- | --- | --- | --- |
| Ahmed | Kumail | Department of Paediatrics and Child Health, Aga Khan University, Karachi, Pakistan | [kumail.ahmed@aku.edu](mailto:kumail.ahmed@aku.edu) |
| Ahmed | Sheraz | Department of Paediatrics and Child Health, Aga Khan University, Karachi, Pakistan | [sheraz.ahmed@aku.edu](mailto:sheraz.ahmed@aku.edu) |
| Alam | Md. Ashraful | Nutrition Research Division, International Centre for Diarrhoeal Disease Research, Bangladesh, Dhaka, Bangladesh | [mashraful@icddrb.org](mailto:mashraful@icddrb.org) |
| Das | Subhasish | Nutrition Research Division, International Centre for Diarrhoeal Disease Research, Bangladesh, Dhaka, Bangladesh | [subhasish.das@icddrb.org](mailto:subhasish.das@icddrb.org) |
| Denson | Lee A. | Division of Pediatric Gastroenterology, Hepatology, and Nutrition, Cincinnati Children's Hospital Medical Center, Cincinnati, OH, USA | [Lee.Denson@cchmc.org](mailto:Lee.Denson@cchmc.org) |
| Fahim | Shah Mohammad | Nutrition Research Division, International Centre for Diarrhoeal Disease Research, Bangladesh, Dhaka, Bangladesh | [mohammad.fahim@icddrb.org](mailto:mohammad.fahim@icddrb.org) |
| Gazi | Md. Amran | Nutrition Research Division, International Centre for Diarrhoeal Disease Research, Bangladesh, Dhaka, Bangladesh | [amran.gazi@icddrb.org](mailto:amran.gazi@icddrb.org) |
| Haberman | Yael | Division of Pediatric Gastroenterology, Hepatology, and Nutrition, Cincinnati Children’s Hospital Medical Center, Cincinnati, OH, USA | [Yael.Haberman@cchmc.org](mailto:Yael.Haberman@cchmc.org) |
| Hasan | Md. Mehedi | Nutrition Research Division, International Centre for Diarrhoeal Disease Research, Bangladesh, Dhaka, Bangladesh | [md.hasan@icddrb.org](mailto:md.hasan@icddrb.org) |
| Hossain | Md. Shabab | Nutrition Research Division, International Centre for Diarrhoeal Disease Research, Bangladesh, Dhaka, Bangladesh | [dr.shabab@icddrb.org](mailto:dr.shabab@icddrb.org) |
| Hotwani | Aneeta | Department of Paediatrics and Child Health, Aga Khan University, Karachi, Pakistan | [aneeta.hotwani@aku.edu](mailto:aneeta.hotwani@aku.edu) |
| Iqbal | Junaid | Department of Paediatrics and Child Health, Aga Khan University, Karachi, Pakistan | [junaid.iqbal@aku.edu](mailto:junaid.iqbal@aku.edu) |
| Iqbal | Najeeha Talat | Department of Paediatrics and Child Health, Aga Khan University, Karachi, Pakistan | najeeha.iqbal@aku.edu |
| Jakhro | Sadaf | Department of Paediatrics and Child Health, Aga Khan University, Karachi, Pakistan | [sadaf.jakhro@aku.edu](mailto:sadaf.jakhro@aku.edu) |
| Kabir | Furqan | Department of Paediatrics and Child Health, Aga Khan University, Karachi, Pakistan | [furqan.kabir@aku.edu](mailto:furqan.kabir@aku.edu) |
| Liu | Ta-Chiang | Department of Pathology and Immunology, Washington University School of Medicine, St. Louis, MO, USA | [ta-chiang.liu@wustl.edu](mailto:ta-chiang.liu@wustl.edu) |
| Mann | Barbara J. | Department of Medicine, University of Virginia School of Medicine, Charlottesville, VA, USA | [bjm2r@virginia.edu](mailto:bjm2r@virginia.edu) |
| Marie | Chelsea | Department of Medicine, University of Virginia School of Medicine, Charlottesville, VA, USA | [csm8r@virginia.edu](mailto:csm8r@virginia.edu) |
| Mazumder | Ramendra Nath | Nutrition Research Division, International Centre for Diarrhoeal Disease Research, Bangladesh, Dhaka, Bangladesh | [ramen@icddrb.org](mailto:ramen@icddrb.org) |
| Mudenda | Victor | Tropical Gastroenterology & Nutrition group, University of Zambia School of Medicine, Lusaka, Zambia | [mudenda2003@yahoo.co.uk](mailto:mudenda2003@yahoo.co.uk) |
| Mulenga | Chola | Tropical Gastroenterology & Nutrition group, University of Zambia School of Medicine, Lusaka, Zambia | [cholamulenga94@gmail.com](mailto:cholamulenga94@gmail.com) |
| Qureshi | Abdul Khalique | Department of Paediatrics and Child Health, Aga Khan University, Karachi, Pakistan | [abdul.qureshi@aku.edu](mailto:abdul.qureshi@aku.edu) |
| Rahman | Masudur | Department of Gastroenterology, Sheikh Russel National Gastroliver Institute and Hospital, Dhaka, Bangladesh | [drmasud47@yahoo.com](mailto:drmasud47@yahoo.com) |
| Rahman | Najeeb | Department of Paediatrics and Child Health, Aga Khan University, Karachi, Pakistan | [najeeb.rahman@aku.edu](mailto:najeeb.rahman@aku.edu) |
| Sadiq | Kamran | Department of Paediatrics and Child Health, Aga Khan University, Karachi, Pakistan | [kamran.sadiq@aku.edu](mailto:kamran.sadiq@aku.edu) |
| Tearney | Guillermo J | Department of Pathology, Harvard Medical School, Boston, MA, USA | [gtearney@partners.org](mailto:gtearney@partners.org) |
| Umrani | Fayaz | Department of Paediatrics and Child Health, Aga Khan University, Karachi, Pakistan | [fayaz.umrani@aku.edu](mailto:fayaz.umrani@aku.edu) |
| Yilmaz | Omer H | Department of Pathology, Massachusetts General Hospital, Boston, MA, USA | [oyilmaz@mgh.harvard.edu](mailto:oyilmaz@mgh.harvard.edu) |
